# Development and Validation of Targeted Metabolomics Methods Using Liquid Chromatography–Tandem Mass Spectrometry (LC-MS/MS) for the Quantification of 235 Plasma Metabolites

**DOI:** 10.3390/molecules30030706

**Published:** 2025-02-05

**Authors:** Kangkang Xu, Franz Berthiller, Barbara U. Metzler-Zebeli, Heidi E. Schwartz-Zimmermann

**Affiliations:** 1 Institute of Bioanalytics and Agro-Metabolomics, Department of Agricultural Sciences, BOKU University, 3430 Tulln, Austria; kangkang.xu@boku.ac.at (K.X.); franz.berthiller@boku.ac.at (F.B.); 2 Christian Doppler Laboratory for Innovative Gut Health Concepts of Livestock, 1210 Vienna, Austria; barbara.metzler@vetmeduni.ac.at; 3 Austrian Competence Centre for Feed and Food Quality, Safety and Innovation (FFoQSI), 3430 Tulln, Austria; 4 Unit Nutritional Physiology, Department of Biomedical Sciences, University of Veterinary Medicine, Veterinaerplatz 1, 1210 Vienna, Austria

**Keywords:** metabolomics, plasma metabolites, targeted HPLC-MS/MS methods, HILIC, reversed-phase high-performance liquid chromatography, animal metabolomics

## Abstract

Plasma contains metabolites with diverse physicochemical properties, ranging from highly polar to highly apolar, and concentrations spanning at least nine orders of magnitude. Plasma metabolome analysis is valuable for monitoring health and evaluating medical interventions but is challenging due to the metabolome’s diversity and complexity. This study aims to develop and validate targeted LC-MS/MS methods for quantifying 235 mammalian metabolites from 17 compound classes in porcine plasma without prior derivatization. Utilizing reversed-phase and hydrophilic interaction liquid chromatography coupled with tandem mass spectrometry, each analyte is identified and quantified using two selected reaction monitoring (SRM) transitions. Fast polarity switching and scheduled SRM enhance the metabolome coverage and throughput, enabling the analysis of one sample in about 40 min. A simple “dilute and shoot” sample preparation protocol was employed, with samples injected at two dilution levels to align metabolite concentrations within calibration curve ranges. Validation in porcine plasma included assessments of carryover, linearity, detection and quantification limits, repeatability and recovery. The method was further applied to plasma samples from various animal species, demonstrating its applicability to human and animal studies. This study establishes two robust LC-MS/MS methods for comprehensive porcine plasma metabolome quantification, advancing large-scale targeted metabolomics in biomedical research.

## 1. Introduction

Metabolomics is the comprehensive study of a wide range of low-molecular-weight compounds, called metabolites, in a biological system. Metabolomics provides a snapshot of the metabolic products of an organism during biological processes. Metabolomics facilitates an enhanced understanding of biochemical and biological mechanisms in intricate systems [1]. It has been commonly utilized as a tool for biomarker discovery and metabolic pathway research [2,3].

Unlike untargeted metabolomics, which aims at measuring all the metabolites in a sample without prior information and provides only relative analyte abundances, targeted metabolomics allows researchers to identify and quantity specific sets of known metabolites [4]. A necessity to determine a wide range of metabolites is the use of a universal detection method, such as mass spectrometry (MS) or nuclear magnetic resonance (NMR). MS- or tandem mass spectrometry (MS/MS)-based metabolomics allows for the profiling of a large number of metabolites over a wide concentration range in a single analysis—a feature that less sensitive metabolomics assays based on NMR cannot compete with. In targeted metabolomics, a triple-quadrupole mass spectrometer (QqQ) is commonly used in selected reaction monitoring (SRM) mode, which is more affordable, sensitive and robust than high-resolution mass spectrometry [5]. Absolute quantification is then realized on the basis of calibration curves established from authentic reference compounds or by comparison with a specified quantity of isotopically labeled internal standards.

Flow injection analysis with tandem mass spectrometry (FIA-MS/MS), one of the formats in targeted metabolomics, works without an analytical column [6,7,8]. The disadvantages of FIA-MS/MS are the occurrence of matrix effects due to the lack of a chromatographic separation step and the potential confusion of isomeric and even isobaric compounds, which can lead to the incorrect identification and quantification of the analyte if no unique fragmentation patterns are available [9,10]. In contrast, the combination of liquid chromatography with tandem mass spectrometry (LC-MS/MS) separates analytes in complex samples efficiently according to the diverse physicochemical properties of the metabolites. This decreases matrix effects, improves limits of detection and quantification and greatly reduces the misidentification of isomeric compounds. In this respect, hydrophilic interaction chromatography (HILIC) has demonstrated its ability to separate polar and charged metabolites in biological matrices, which are hardly retained in reversed-phase liquid chromatography (RPLC) [11,12,13]. As one single analytical method is insufficient to cover the entire metabolome, a combination of chromatographic separation methods can significantly increase metabolite coverage [14,15].

Metabolomics analysis has been extensively applied to study biofluids like blood, plasma, serum, saliva and urine, as reviewed in [16,17]. The blood metabolome is used to evaluate and monitor internal and external perturbations of an organism’s metabolism [18,19], providing insights into its physiological and health conditions [20]. Plasma is the supernatant obtained by centrifuging blood that has been treated with an anticoagulant (e.g., EDTA, sodium citrate or heparin) to prevent clotting, thereby separating it from the cellular components. Plasma comprises metabolites with diverse physicochemical properties, from highly polar to highly apolar, and their concentrations span nine orders of magnitude [21,22,23,24]. The complexity of the plasma metabolome poses a challenge for the choice of an analysis platform. In recent years, large-scale targeted metabolomics methods for the quantification of hundreds of human or rodent plasma metabolites from diverse compound classes were established, e.g., [13,25,26,27,28,29]. For instance, McMillen et al. [27] developed two large-scale, targeted LC-MS/MS methods based on RPLC and HILIC, respectively, to (semi)quantify 540 metabolites in human serum. Likewise, Lee and co-workers [26] presented two RPLC-MS/MS and HILIC-MS/MS methods for the quantification of over 200 metabolites related to core metabolic pathways in a wide range of biological samples, including serum, tissues and cells from humans and mice. Li et al. [13], Cao et al. [25] and Nandania and co-workers [29] developed and used single HILIC-MS/MS methods for the quantitative determination of 610 [13], 428 [25] and 102 [29] metabolites in various biological matrices including plasma or serum. Wei et al. [28] determined 205 polar metabolites in rat plasma by the parallel use of three analytical columns with different separation mechanisms and using a sophisticated system of sequential elution and MS/MS detection. However, most of these assays show notable limitations, such as lack of certain validation parameters or poor validation results [27]. Apparent recoveries that are crucial for accurate quantification were provided only by McMillen et al. [27] and Nandania and co-workers [29]. Limits of detection and quantification were only stated by McMillen [27], Wei et al. [28] and Cao et al. [25], and only partly for a selected number of analytes (96 metabolites for [27], 20 amino acids for [25]). Only Cao et al. [25] and Lee et al. [26] provided two SRM transitions for each analyte to ensure correct compound identification. Additionally, to date, there are no fully validated LC-MS/MS-based targeted metabolomics methods available for porcine plasma. Instead, ready-to-use commercial metabolomic kits relying on the derivatization of amino acids and amines have been widely utilized for the quantification of porcine plasma metabolites, e.g., [20,30,31,32,33]. In addition, a set of three RP-HPLC-MS/MS methods was developed for the quantitative determination of 176 porcine metabolites from various compound classes. These methods also employed the derivatization of amino acids, amino acid-related compounds and biogenic amines to improve the ionization and separation of analytes [34] but were not validated for porcine plasma. Moreover, the derivatization step prolongs and complicates sample preparation, which can lead to impaired repeatability. Likewise, some of the derivatized analytes have limited stability, necessitating immediate analysis or the repetition of sample work-up. Another common challenge in targeted LC-MS/MS methods is managing the trade-off between analysis time and metabolome coverage. Multiple methods are required to achieve a high metabolome coverage, which prolongs the analysis time and increases analysis costs.

In this study, we aim to develop and thoroughly validate two targeted multi-analyte LC-MS/MS methods for the determination of 235 mammalian metabolites from 17 compound classes in porcine plasma samples. Both RPLC and HILIC coupled to QqQ-MS will be used after simple sample preparation without derivatization. To further improve the correct identification of analytes, two SRM transitions (a quantifier and a qualifier) are to be measured. Fast polarity switching and scheduled SRM (sSRM) will be employed to simultaneously quantify metabolites in both polarities. The analytical performance of these methods, including limits of detection and quantification, linearity, repeatability, apparent recovery, carryover and trueness, will be determined. Furthermore, we will test the applicability of the methods to plasma samples from other species. Our large-scale targeted metabolomics methods will overcome current limitations in porcine plasma metabolomics, including metabolite misidentification, speed of analysis and accuracy of quantification.

## 2. Results and Discussion

### 2.1. Method Development

#### 2.1.1. Targeted Metabolites

**Figure 1:**
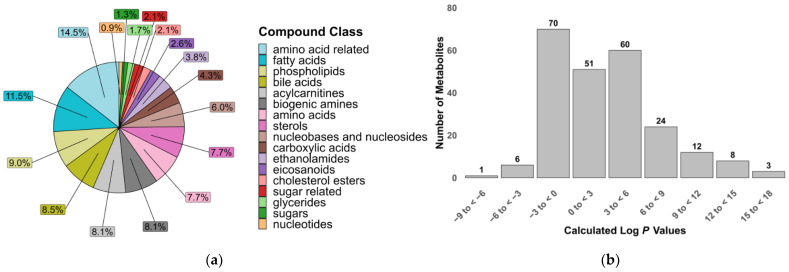
Properties of metabolites covered in the targeted metabolomics assay. (**a**) Relative proportion of compound classes; (**b**) calculated octanol–water partition coefficients (log *p*) of 235 metabolites.

A total of 235 metabolites were included in our two LC-MS/MS methods, selected based on the commercial availability of standards and their suitability for reliable quantification under LC conditions. The metabolites were grouped into 17 compound classes of biological significance to human and animal health, with a particular focus on inflammation and gut dysbiosis. These classes included polar compounds such as amino acids, sugars and biogenic amines, as well as signaling lipids like fatty acids, phospholipids and sterols [34,35]. The physicochemical diversity of the metabolites in this assay is demonstrated by the range of calculated log *p* values that partially reflect the compounds’ lipophilicity (Figure 1).

The initial assignment of metabolites to an LC method was based on retention factors determined in our preceding study [14]. Metabolites with a retention factor > 1 in the bare-silica HILIC method were chosen for HILIC method development, while those well retained in the RP-C18 method or under lipidomics conditions were selected for the RP method. Metabolites potentially suitable for both methods were originally measured under both conditions. Eventually, metabolites detected in both polarities and/or with both methods were assigned to the method and polarity in which they showed the highest signal intensity. With only a few exceptions, each metabolite was measured only in one polarity per method.

#### 2.1.2. LC Conditions

One of the major challenges in plasma metabolomics is separating highly diverse classes of metabolites, which cannot be achieved using just a single chromatographic method. Therefore, we initially selected the RP-C18 method and the bare-silica HILIC method from our previous study to enhance the metabolic coverage [14]. Mobile phase composition, flow rate and column oven temperature were modified to achieve optimal separation performance. To minimize the potential carryover of highly hydrophobic lipids, the solvent strength of mobile phase B in the RP method was increased by replacing pure acetonitrile (ACN) with 47.5/47.5/5 isopropanol (IPA)/ACN/water (*v*/*v*/*v*). Additionally, the isocratic wash period of the RP run was extended to 11.5 min. To enhance the removal of highly polar compounds in the HILIC method, the percentage of the aqueous mobile phase was increased from the original 40% to 60% in the isocratic wash period. Furthermore, the flow rate was doubled during the wash periods to accelerate the methods, while the oven temperature was increased to ensure that the column pressure remained within an acceptable range. To avoid issues like pump pressure fluctuations, the organic mobile phase of both methods contained 5% water, ensuring the solubility of solvent additives.

**Figure 2:**
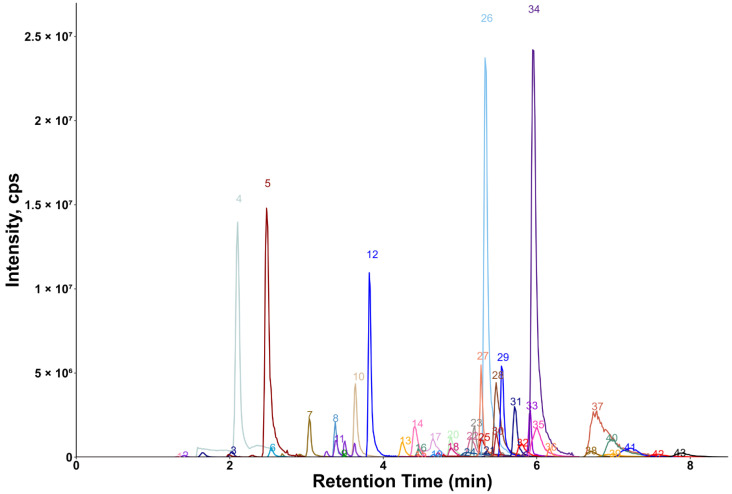
Extracted ion chromatogram of the QC plasma sample measured by HILIC-MS/MS. The peak intensity of betaine (peak 34) was scaled (divided by 3) for better visualization. 1: 2′-deoxythymidine; 2: uridine; 3: xanthine; 4: urea; 5: hypoxanthine; 6: inosine; 7: guanine; 8: cytidine; 9: 2′-deoxycytidine; 10: taurine; 11: cytosine; 12: creatinine; 13: tryptophan; 14: phenylalanine; 15: pyroglutamic acid; 16: tyrosine; 17: leucine; 18: pyrrolidine; 19: methionine; 20: valine; 21: N-methylalanine; 22: threonine; 23: alanine; 24: glutamic acid; 25: trans-4-hydroxyproline; 26: proline; 27: trimethylamine-N-oxide; 28: glutamine; 29: creatine; 30: propionylcarnitine; 31: acetylcarnitine; 32: citrulline; 33: trigonelline; 34: betaine; 35: carnitine; 36: stachydrine; 37: arginine; 38: histamine; 39: ornithine; 40: lysine; 41: N,N-dimethylarginine; 42: 3-methylhistidine; 43: 1-methylhistidine.

**Figure 3:**
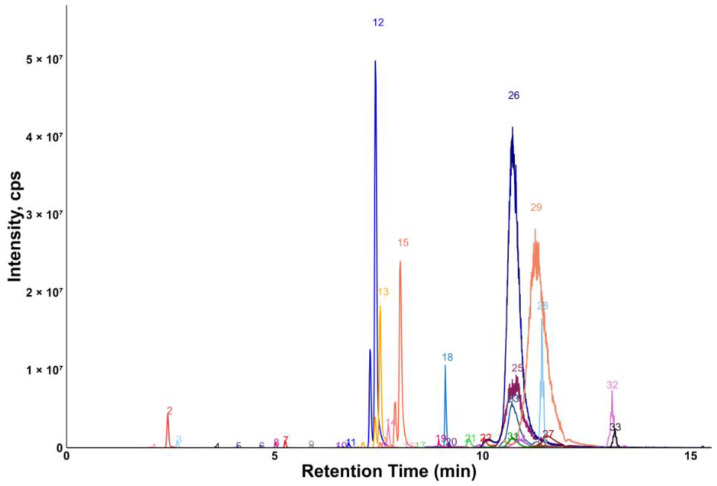
Extracted ion chromatogram of the QC plasma sample measured by RP-MS/MS. 1: 3-indoxylsulfate; 2: hippuric acid; 3: phenylacetylglycine; 4: 3-indoleacetic acid; 5: 3-indolepropionic acid; 6: taurohyodeoxycholic acid; 7: glycoursodeoxycholic acid; 8: glycocholic acid; 9: glycochenodeoxycholic acid; 10: chenodeoxycholic acid; 11: 1-myristoyl-2-hydroxy-sn-glycero-3-phosphatidylcholine; 12: 1-palmitoyl-2-hydroxy-sn-glycero-3-phosphatidylcholine; 13: 1-oleoyl-2-hydroxy-sn-glycero-3-phosphatidylcholine; 14: 1-heptadecanoyl-2-hydroxy-sn-glycero-3-phosphatidylcholine; 15: 1-stearoyl-2-hydroxy-sn-glycero-3-phosphatidylcholine; 16: linoleic acid; 17: oleic acid; 18: cholesterol; 19: 24α-methylcholesterol; 20: N-palmitoyl-D-sphingosin; 21: 1,2-dipalmitelaidoyl-sn-glycero-3-phosphatidylcholine; 22: 1-palmitoyl-2-myristoyl-sn-glycero-3-phosphatidylcholine; 23: 1-oleoyl-2-stearoyl-sn-glycero-3-phosphatidylcholine; 24: 1-O-hexadecyl-2-arachidonoyl-sn-glycero-3-phosphatidylcholine; 25: C16 sphingomyelin; 26: 1-stearoyl-2-arachidonoyl-sn-glycero-3-phosphatidylcholine; 27: 1,2-distearoyl-sn-glycero-3-phosphatidylcholine; 28: 1,2-dilinoleoyl-3-palmitoylglycerol; 29: 1,2-dielaidoyl-sn-glycero-3-phosphatidylcholine; 30: 1-1(Z)-hexadecenyl-2-palmitoyl-sn-glycero-3-phosphatidylcholine; 31: C16-18:1 PC; 32: 1,3-dioleyl-2-palmitoylglycerol; 33: 1,3-dipalmitoyl-2-oleoylglycerol.

Chromatograms showing the majority of the metabolites detected in the porcine quality control (QC) plasma sample are shown in Figure 2 (HILIC) and Figure 3 (RP). The metabolites analyzed by HILIC-MS/MS were eluted mainly between 1 and 8 min, whereas the compounds targeted by the RP method were eluted within the gradient from 2 to 15 min. Notably, seven phospholipids had broader peaks, as exemplified by peak 26 and peak 29 in Figure 3. As discussed in our previous study [14], we observed similarly broad peaks for other phosphorylated metabolites, such as nucleotides and sugar phosphates. This can be explained by the partial adsorption of phosphate-containing analytes onto metal surfaces within the LC-ESI-MS system, such as stainless-steel components used in the injector, column housing, tubing, connectors and similar parts. Additionally, these seven phospholipids had slightly different retention times in porcine plasma compared to those in pure solvent standards. This indicates the presence of isomers with different double bond positions in the samples, which caused retention time shifts. Therefore, the detection window for these seven metabolites was increased to 60 s to account for this variability.

A significant source of interference arises from isomeric or isobaric metabolites that exhibit similar chromatographic behaviors and potentially have similar primary fragmentation patterns [28]. The separation of these compounds, while concurrently resolving a broad spectrum of other metabolites, represents an inherent challenge in targeted metabolomics. Remarkably, certain isomeric metabolites can be effectively baseline separated. For instance, α-aminobutyric acid (AABA), β-aminobutyric acid (BABA) and γ-aminobutyric acid (GABA) represent three isomers of aminobutyric acids, which were independently eluted with retention time differences of 0.05 min and 0.22 min between each pair. An LC discrimination between symmetric N,N′-dimethylarginine (SDMA) and asymmetric N,N-dimethylarginine (ADMA) was also achieved. Other isomeric pairs, e.g., valine and norvaline, were partially separated, requiring deconvolution for peak integration. In contrast, leucine and isoleucine could not be separated in our HILIC method. Instead, they were differentiated by their specific MS/MS fragmentation patterns. The unique transition of *m/z* 132.1 → 69 enabled the quantification of isoleucine, while another transition, *m/z* 132.1 → 89, where both compounds had similar peak areas at equal concentrations, was used to estimate the sum concentration of leucine and isoleucine. The leucine concentration was then calculated by subtracting the isoleucine concentration from the sum concentration.

Nonetheless, certain isobaric compounds remained unresolved in the current methods. For example, hexoses, pentoses, alanine, β-alanine and sarcosine could be separated neither chromatographically nor by mass spectrometry. Consequently, the provided concentrations of hexoses, pentoses and alanine isobaric compounds are sum concentrations.

#### 2.1.3. MS/MS Conditions

Our methods use two SRM transitions (quantifier and qualifier), enabling the calculation of the ion ratio, which can be used with the retention time for unambiguous compound identification. The best-suited quantifier and qualifier transitions were selected by measuring authentic standards with both LC methods in both polarities using all SRM transitions provided in our previous work [14]. For each metabolite, the two SRM transitions showing the highest signal-to-noise ratios were chosen for the final methods. Exceptions included cholesteryl myristate, cholesteryl palmitoleate and 3-hydroxyphenylacetic acid, for which only one reliable SRM transition was available. Additionally, six metabolites, namely serine, myo-inositol and four bile acids, were measured in both polarities because it was impossible to identify two reliable SRM transitions in a single polarity. As a result, a total of 467 SRM transitions were incorporated into our targeted LC-MS/MS methods.

We applied fast polarity switching in our LC-MS/MS methods to measure in both positive and negative polarity within one run, reducing two separate injections to one. However, impaired repeatability is a common concern when simultaneously targeting a large number of metabolites in both negative and positive ion modes. This is attributed to fewer data points within one chromatographic peak when accelerating large-scale targeted metabolomics assays, which in return diminish accurate and precise quantification [36]. This disadvantage was tackled by further employing scheduled SRM, where each SRM transition is monitored within a narrow retention time window to ensure an optimal dwell time and number of data points for higher levels of SRM multiplexing [37].

**Figure 4:**
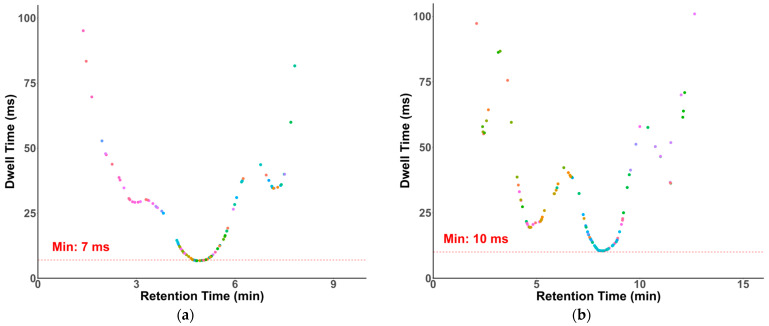
Dwell time plots. (**a**) HILIC-MS/MS with a minimal dwell time of 7 ms; (**b**) RP-MS/MS with a minimal dwell time of 10 ms.

Dwell times in our methods were optimized and generated by SciexOS. Modern QqQ instruments have demonstrated reliable quantification with dwell times as short as 3 ms [38]. As shown in Figure 4, despite a large number of compounds eluting around 5 min and 8 min, the lowest dwell times of the transitions in our HILIC and RP methods were 7 and 10 ms, respectively.

### 2.2. Method Validation

#### 2.2.1. Linearity

The determination of the limits of detection (LODs), lower limits of quantification (LLOQs) and upper limits of quantification (ULOQs) was conducted using 14 calibration levels of standard solutions. Analytes with poor linearity (extremely high LLOQs) due to weak ionization efficiency were excluded from our methods. Examples of these compounds included keto- and aromatic carboxylic acids and several cholesterol esters.

The linear range varied among metabolites, with 64% of the metabolites exhibiting a wide linear response over two orders of magnitude, compared to 33% with a narrower linear range of one to two orders of magnitude. Metabolites displaying linearity below one order of magnitude, for instance dopamine, dehydroergosterol and myristic acid, also possessed high LLOQs. Overall, our method demonstrated good linearity for most compounds. Moreover, 99% of the porcine metabolites quantified in the applicability test had a coefficient of determination (r^2^) exceeding 0.900; 64% of the quantified porcine metabolites showed a coefficient of determination ≥ 0.995; and 30% had a coefficient of determination ≥ 0.999.

#### 2.2.2. Carryover

Carryover can adversely impact the accurate qualitative and quantitative determination of compounds and may restrict the dynamic range of quantitation methods based on LC-MS [39]. The calculated carryover percentage for each analyte is provided in Table S1. In total, 203 out of 235 metabolites had a carryover rate of less than 5%, of which 152 metabolites exhibited a carryover below 1%. Only 6% of the metabolites covered by HILIC and RP, respectively, exceeded 5% carryover. Notably, the majority of metabolites with high carryover in the RP method were long-chain fatty acids, such as oleic acid and arachidic acid. The generally low carryover can be attributed to our systematic approach to counteracting analyte adsorption, including a rigorous autosampler cleaning plan involving washes with multiple column volumes of strong eluent at the end of the gradient and blank injections throughout the sequence.

#### 2.2.3. Recovery

Recovery experiments, aimed at evaluating analyte losses during sample preparation in addition to assessing mass spectrometric matrix effects, involve adding defined amounts of standard compounds to the sample prior to work-up [40]. Apparent recoveries (R_As_) were assessed by the standard addition method, which partly requires spiking very high concentrations of metabolites into plasma samples. The limited solubility of some compounds makes it impossible to simply spike solutions into plasma samples without significantly diluting the sample. As such, for method validation only, stock solutions had to be evaporated before taking up metabolites in plasma or pure solvents. Thus, we comprehensively tested various solvent mixtures with decreasing polarity for the uptake of RP metabolites, namely ACN/water (80/20, *v*/*v*, polarity index = 6.8), methanol (MeOH) (polarity index = 6.6), ACN (polarity index = 6.2), acetone/water (80/20, *v*/*v*, polarity index = 6.1), ACN/IPA (50/50, *v*/*v*, polarity index = 5.3), IPA/water (80/20, *v*/*v*, polarity index = 5.2), acetone/IPA (50/50, *v*/*v*, polarity index = 4.9) and pure IPA (polarity index = 4.3) (detailed results not shown). IPA/water (80/20, *v*/*v*) yielded the highest recovery of 91% of total RP metabolites. To verify if IPA/water (80/20, *v*/*v*) was also suitable for the uptake of HILIC compounds, it was compared to ACN/water (80/20, *v*/*v*), which is routinely used as extraction solvent for HILIC metabolites. As both solvent mixtures yielded similar results, IPA/water (80/20, *v*/*v*) was used as the only extraction solvent for the work-up of samples and for the validation experiments for both methods.

The standard addition protocol was applied to all 235 metabolites, including those that were not detected in our QC porcine plasma sample. This approach was chosen because any of these metabolites could potentially occur in plasma and serve as biomarker, depending on the design of animal trials and/or the animal species analyzed. The standard addition plan involved spiking six levels into porcine QC plasma in triplicate. This, however, was impossible for certain undetected metabolites exhibiting high LLOQs, primarily hydrophobic fatty acids, cholesterol ester and sterols. For these 31 metabolites, only one high level was spiked into plasma, as excessive concentrations could negatively impact solubility and subsequent reconstitution. Recovery data for both dilutions of plasma (1:5, 1:100, *v*:*v*) are presented in Table S2.

Recovery rates between 70% and 130% are considered acceptable [40]. Any deviations outside of the acceptable range indicate that a metabolite requires further investigation to ensure accurate quantification. Metabolites showing low and inconsistent R_As_ were eliminated from our methods. For instance, five hydrophobic metabolites (e.g., 1,2-dioleoyl-sn-glycerol, cholesteryl arachidonate and C18 galactosyl(β) ceramide) originating from glycerides, cholesterol esters and phospholipids exhibited poor and erratic linearity in both spiked samples and standards in pure solvent solutions. Low and inconsistent recoveries of certain metabolites, particularly associated with hydrophobic compounds, pose a challenge for LC-MS/MS method development and validation [41]. These issues arise from analyte losses during sample preparation and/or from matrix effects during analysis [41]. Furthermore, no single pre-treatment adequately covers the entire metabolome [42]. In particular, large-scale LC-MS/MS methods target diverse metabolite classes with varied physio-chemical properties, and one single set of sample preparation could compromise the recoveries of some analytes. This can be exemplified by cholesteryl arachidate and C18 galactosyl(β) ceramide. Both had a poor recovery of 49% and 208%, respectively, using IPA/water (80/20, *v*/*v*) as the extraction solvent and therefore yielded low or variable R_As_.

**Figure 5:**
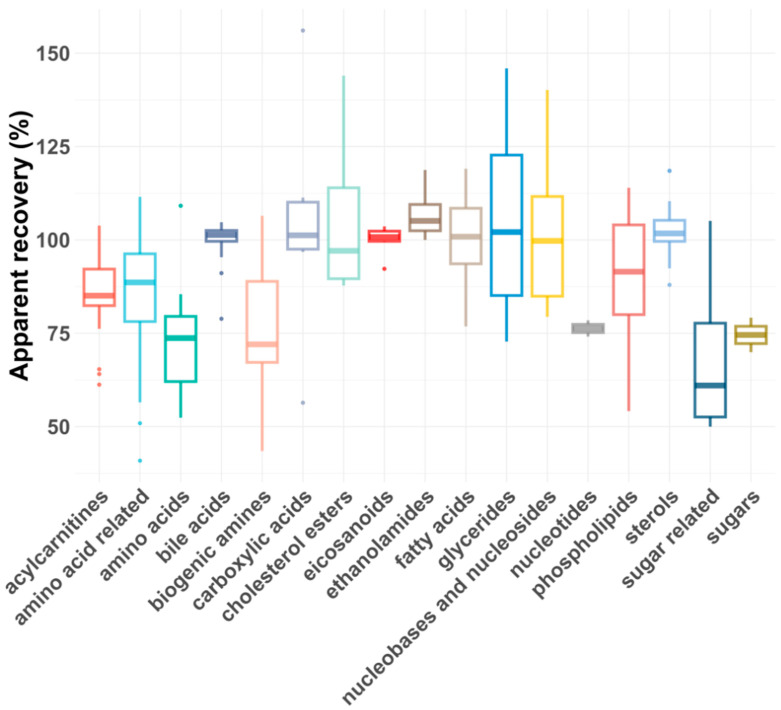
Box plot showing apparent recoveries of metabolite classes for 1:5 dilution.

**Figure 6:**
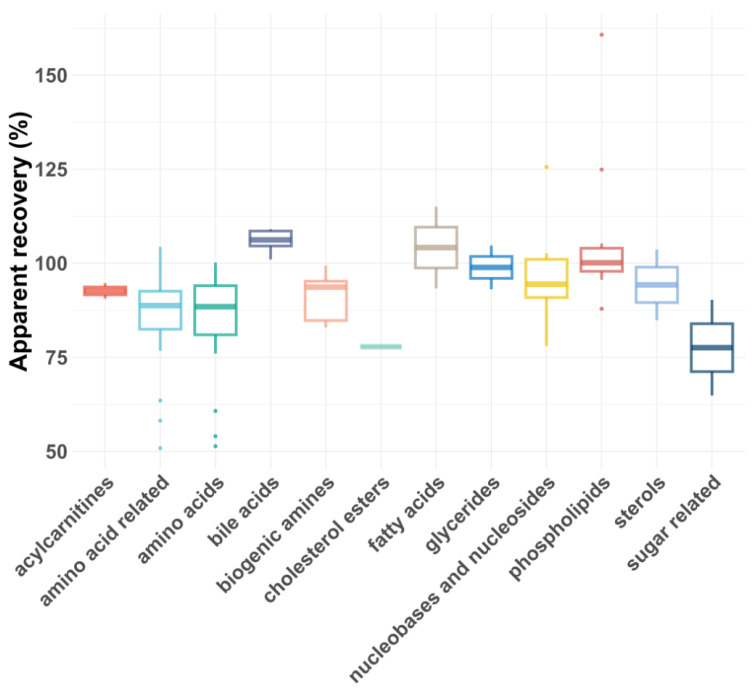
Box plot showing apparent recoveries of metabolite classes for 1:100 dilution.

As shown in Figure 5 and Figure 6, the average R_As_ across all compound classes met the acceptance criteria, regardless of plasma dilution. However, single analytes had lower or, rarely, higher R_As_, most likely due to matrix effects. In general, matrix effects can be minimized by injecting smaller volumes or diluting the samples, provided the compounds are still above the LLOQ [43]. For five compound classes (carboxylic acids, eicosanoids, ethanolamides, nucleotides and sugars), only the 1:5 plasma dilution yielded reliable apparent recoveries, as a 1:100 dilution reduced their concentrations below the LLOQs. For certain compound classes where both plasma dilutions were feasible, the R_As_ and intra-class consistency improved upon 1:100 dilution, presumably due to reduced matrix effects. For example, the average recovery of biogenic amines increased from 77% to 91%, and the average recovery of amino acids improved from 72 to 83%. Additionally, the coefficient of variation (CV%) decreased significantly for some compound classes after dilution, with glycerides improving from 30% to 8% and biogenic amines from 22% to 7%.

Glucose was excluded from the 1:100 dilution analysis due to its high concentration in QC plasma (>400 mg/L, estimated by 1:500 dilution) and relatively low ULOQ (3 mg/L in measurement solution). Despite the dilution (1:100, *v*:*v*), glucose had an R_A_ of only 29% with poor repeatability (105%). Because of the high plasma concentration and the poor validation results at a 1:100 dilution, glucose was excluded from the method. A 1:500 dilution would be required for the validation and accurate quantification of glucose.

#### 2.2.4. Repeatability

Repeatability was expressed as the relative standard deviation (RSD) of the recoveries determined at six spiking levels in triplicate (*n* = 18). A total of 94% of metabolites exhibited consistent RSD values of ≤25%. Previous studies have linked poor repeatability or reproducibility to low peak intensity, low recovery or poor chromatography [28,29]. This aligns with our findings for some metabolites with high RSD values, such as cholesteryl docosapentaenoate and C24 dihydroceramide (d18:0/24:0), showing low peak intensity and low recovery despite high spiking concentrations.

#### 2.2.5. Trueness and Applicability to Plasma from Other Animal Species

Due to the absence of a certified reference material for porcine plasma, the human plasma NIST standard reference material (SRM) 1950 was analyzed as a surrogate to assess the trueness of the method, given the physiological similarities of the two species. The concentrations obtained by our method were in line with the NIST reference values. Overall, the concentrations of 11 out of 15 NIST metabolites were within 70–130% of the certified values, demonstrating the excellent trueness of our analytical approach (Table 1).

Both cholecalciferol and calciferol were below the LODs in our methods. These compounds had low concentrations in the NIST reference sample, where dedicated methods had been used for quantification. Glycine was removed from our HILIC method due to a significant mismatch with the NIST reference values. The NIST value calculated by our HILIC method was eleven times higher than the reference value. As glycine did not fragment in MS/MS analysis, two “pseudo-molecular” SRM transitions were selected, with SRM transitions from the protonated/deprotonated precursor ions to the respective precursor ions. The non-specific SRM transitions could be more susceptible to matrix interference, leading to an overestimation of the measured values in the NIST sample.

In total, our methods quantified 124 out of 235 metabolites in the QC porcine plasma sample. Besides porcine plasma, the method applicability was also tested on plasma from other species, including human, rat, bovine and chicken plasma. A comprehensive compilation of metabolite concentrations is reported in Table S3. Overall, we quantified 159 out of 235 metabolites in the analyzed human and animal plasma samples. Among the 76 metabolites that were not detected, 30 possessed an LOQ ≤ 30 µg/L in measurement solution, indicating at best only low concentrations of these metabolites in plasma. Moreover, 46 undetected metabolites, primarily keto- and aromatic carboxylic acids, fatty acids, cholesterol esters and sterols, showed higher LOQs, most likely due to mediocre ionization under the electrospray conditions used. However, these undetected metabolites were still kept in our methods, as they may be present in larger quantities and serve as potential biomarkers depending on the design of the animal trial and/or the animal species. Additionally, the use of another ionization technique like atmospheric pressure chemical ionization or of a different mass spectrometer with a different ion source may result in improved LODs and LOQs. To address the challenge of high LODs of compounds naturally present in low concentrations, injecting larger volumes or utilizing different ionization techniques or derivatization could be viable alternatives.

The pattern of plasma metabolites differed between animal species, which highlights differences in metabolic pathways among species. One notable example is the presence of urea: while mammals like pigs, humans, rats and cattle excrete nitrogen primarily as urea, chickens predominantly excrete nitrogenous end products as uric acid due to their unique nitrogen metabolism [44]. Furthermore, chickens were found to have lower concentrations of plasma creatinine compared to mammals. The difference arises because birds excrete creatine without its conversion to creatinine, leading to reduced plasma creatinine levels [45]. In general, our compilation of plasma metabolite concentrations serves as a valuable reference for the validation and quantification of metabolites in plasma from diverse animal sources.

#### 2.2.6. Comparison to Other Large-Scale Targeted Metabolomics Methods

Given the complexity of porcine plasma, one analytical method is insufficient to cover the entire metabolome. Using one single LC technique for targeted large-scale metabolomics studies results in poor peak shapes and the loss of some compounds [13]. If only RP is used, additional sample preparation steps are required to further improve the metabolomic coverage, i.e., the derivatization of polar compound classes like carboxylic acids [46,47], amino acids, biogenic amines [34,46]. This in turn complicates the workflow and compromises the reproducibility. To enhance the metabolic coverage, we developed and combined two complementary LC-MS/MS methods: one RP and one HILIC method. Fast polarity switching and sSRM were employed to accelerate the methods and increase the throughput for multi-analyte analysis. The sample preparation was optimized as a simple “dilute and shoot” approach.

Many studies or commercially available metabolomics kits acquire only one SRM transition per analyte, e.g., [13,27,28,29]. This approach carries the risk of interference from a confounding isobaric compound that produces isobaric fragment ions. To counteract the limitation, high-quality targeted metabolomics assays demand at least two transitions for accurate identification and quantitation [48]. In our methods, the two best transitions for each metabolite were carefully selected to minimize the risk of false positives. Retention times, as well as ion ratios (qualifier to quantifier), are given in Table S4 for further confirmation.

Notably, some studies either omitted essential validation parameters, such as recovery data [13,25,26,28], LOD or LOQ [13,26,29], or reported calibration curves with poor correlation coefficients [27]. Without R_A_ data, analyte losses cannot be assessed and, if required, corrected, which affects the accurate quantification of metabolites. LODs and LOQs are essential to assess a method’s capacity to detect and precisely quantify low levels of analytes, while poor linearity remains a primary source of quantification errors [49]. In contrast, our methods underwent a comprehensive validation protocol, demonstrating minimal carryover, excellent linearity, good repeatability and high accuracy.

Additionally, our methods are more affordable and more robust compared to commercial metabolomic kits, which mostly rely on the derivatization of amino acids, amino acid-related compounds and biogenic amines. As our methods include only transitions of compounds available as reference standards to ensure correct quantification, they cover a smaller metabolite number compared to other large-scale plasma metabolomics studies [13,26,27]. Expanding the metabolic coverage by introducing a third LC method, such as anion-exchange chromatography, could improve the detection of gut-related carboxylic acids and phosphate-containing metabolites like sugar phosphate or nucleotides, but this is out of the scope of the presented study.

## 3. Materials and Methods

### 3.1. Chemicals

ACN (LC-MS grade) was purchased from VWR International GmbH (Vienna, Austria) and both IPA (LC-MS grade) and formic acid (FA) (MS grade) from Honeywell (Vienna, Austria). Acetone was obtained from Sigma-Aldrich (Vienna, Austria), while heptane was purchased from J.T. Baker (Deventer, The Netherlands). Aqueous ammonium hydroxide solution (25%, MS grade) and MeOH (LC-MS grade) were procured from Merck (Darmstadt, Germany). LC-MS-grade water was produced using a Arium^®^ Pro water purification system (Sartorius, Göttingen, Germany).

A detailed list providing CAS numbers, sum formulas, SMILES codes, exact masses, log *p* values and providers of the compounds used in this study is provided in Table S5. Stock solutions were prepared mainly at a concentration of 1000 mg/L and stored at −80 °C.

### 3.2. Plasma Samples

Porcine plasma was obtained from the pig facility (Vetfarm) of the University of Veterinary Medicine Vienna. The samples were collected from 48 weaned pigs (Large White × Piétrain, 24 males and 24 females) on day 14 after weaning (on days 39 to 42 of life). Prior to blood sampling, the piglets were anesthetized into the ear vein with azaperone (Stresnil 40 mg/mL, Elanco Tiergesundheit AG, Basel, Switzerland) and ketamine (Narketan 100 mg/mL, Vetoquinol Österreich GmbH, Vienna, Austria). Blood samples were collected by cardiac puncture into plasma tubes (Vacuette^®^ Röhrchen K3E K3EDTA; Greiner Bio-One International GmbH, Kremsmünster, Austria), which were then inverted and stored on ice until centrifugation at 3000× *g* for 20 min at 4 °C (centrifuge 5810 R, Eppendorf, Hamburg, Germany). The obtained plasma was aliquoted and stored at −80 °C until analysis.

A QC plasma sample for method validation was prepared by pooling equal aliquots of all 48 plasma samples. Human, rat, bovine and chicken plasma was purchased from Sigma-Aldrich (Vienna, Austria) as lyophilized powder and reconstituted in the indicated volume of purified water. In addition, SRM 1950–Metabolites in Frozen Human Plasma, originating from the National Institute of Standards and Technology (NIST, Portland, OR, USA), was obtained as a certified reference material to test the method trueness.

### 3.3. Work-Up of Plasma Samples

Sample preparation consisted of protein precipitation by mixing 200 µL of plasma with 800 µL of cold (4 °C) IPA and centrifuging at 14,350× *g* and 4 °C for 10 min. The supernatants were further diluted 1 + 19 (*v* + *v*) with IPA/water (80/20, *v*/*v*). Both samples (1:5 and 1:100 dilution in total) were either measured directly or stored at −80 °C before LC-MS/MS measurement.

### 3.4. Preparation of Calibration Standards

Three sets of standards were prepared to establish calibration curves. For all standard sets, standard mixes were prepared by dilution from the original 1000 mg/L solutions. For all three mixes, 7 concentration levels were prepared that covered up to three orders of magnitude for each individual compound. The actual concentrations of the individual compounds were selected based on the LLOQ, ULOQ (see Table S1), and, following preliminary measurements of porcine plasma samples, the expected concentration range in porcine plasma. For HILIC compounds, the lowest calibration level was 0.03 µg/L, while the highest calibration level was 25,000 µg/L. Due to the limited solubility of some RP compounds, two sets of standards had to be prepared for RP measurements. Set RP_Aqu_ contained polar to mid-polar compounds, while set RP_Hep_ contained mid-polar to nonpolar substances. The sets used are indicated in Table S1. The lowest calibration level in RP mixes was 0.1 µg/L, while the highest level was 50,000 µg/L.

### 3.5. LC-MS/MS Analysis

LC-MS/MS analyses were carried out on an Agilent 1290 Infinity II series UHPLC system (Waldbronn, Germany) coupled to a 6500 triple-quadrupole mass spectrometer equipped with an IonDrive Turbo V^®^ source (Sciex, Foster City, CA, USA).

RP chromatography was carried out on a Kinetex C18 column (150 × 2.1 mm, 2.6 μm, Phenomenex, Aschaffenburg, Germany). The RP column was protected by a SecurityGuard ULTRA pre-column of the same stationary phase (Phenomenex). For RP, mobile phase A consisted of water modified with 1 mM ammonia and 0.1% FA (*v*/*v*), while mobile phase B consisted of 47.5/47.5/5 IPA/ACN/water (*v*/*v*/*v*), also modified with 1 mM ammonia and 0.1% FA (*v*/*v*). The gradient elution started with 5% solvent B from 0.0 to 0.5 min, continued with a linear increase to 100% B between 0.5 and 8.0 min and an isocratic wash period at 100% B between 8.0 and 19.5 min and ended with re-equilibration at 5% B from 19.5 to 23.0 min. The flow rate was 400 µL/min, except during the wash phase of 100% B between 9.5 and 19.5 min, where the flow rate was increased to 800 µL/min to shorten the wash-out period. The injection volume was 1 µL, and separations were carried out at 50 °C for RP.

For HILIC measurements, a Kinetex HILIC column (150 × 2.1 mm, 2.6 μm, Phenomenex) protected by a SecurityGuard ULTRA pre-column of the same stationary phase was used. For HILIC, mobile phase A consisted of 95/5 ACN/water (*v*/*v*) modified with 1 mM ammonia and 0.1% FA (*v*/*v*), while mobile phase B was pure water modified with 1 mM ammonia and 0.1% FA (*v*/*v*). The gradient for the HILIC method was as follows: 0.0–0.5 min: 100% A, 0.5–6.5 min: linear decrease to 63.2% A (60% ACN), 6.5–6.8 min: linear decrease to 42.1% A, 6.8–12.0 min: 42.1% A (40% ACN), 12.0 to 12.8 min: linear increase to 100% A, 12.8–16.5 min: 100% A. The flow rate was 400 µL/min, except during the re-equilibration phase of 100% A between 13.3 and 15.8 min, where the flow rate was increased to 1000 µL/min to accelerate column re-equilibration. The injection volume was 1 µL, and separations were carried out at 40 °C.

Mass spectrometric detection was conducted in sSRM mode using fast polarity switching. The electrospray ionization parameters were electrospray voltage 4500 V for positive mode and −4500 V for negative mode, source temperature 450 °C, curtain gas 30 psi and ion source gases 1 and 2 at 50 psi each. The settling time for polarity switching was 15 ms. The target cycle time was 1000 ms, and the pause time was 5 ms. The detection window width was mainly 30 s in both polarity modes. sSRM transitions, retention times, detection windows, calculated dwell times and ion ratios are shown in Table S4, the data for which can be directly copied into SciexOS (version 3.0.0.3339, Sciex, Foster City, CA, USA).

### 3.6. Data Processing

SciexOS was used for peak integration and to establish linear or quadratic 1/x weighted calibration curves. Peak areas were used for quantification. Instrument drift was corrected by using bracket calibration curves or by normalizing with QC samples throughout the sequence. Statistical analysis and plot generation were performed in MS Excel 2019 (Microsoft, Redmond, WA, USA) and in R 4.2.2 (Bell Laboratories, Murray Hill, NJ, USA).

### 3.7. Method Validation

#### 3.7.1. Spiking Experiments

A preliminary experiment was carried out with 2 mg/L metabolite mixtures in order to determine (a) the potential losses of metabolites during evaporation to dryness (e.g., stability, volatility) and (b) whether compounds could be completely dissolved after evaporation. Three sets of standards (HILIC, RP_Aqu_ and RP_Hep_) were individually evaporated under a stream of nitrogen and reconstituted with various solvents. The tested solvents were ACN/water (80/20, *v*/*v*), MeOH, ACN, acetone/water (80/20, *v*/*v*), ACN/IPA (50/50, *v*/*v*), IPA/water (80/20, *v*/*v*), acetone/IPA (50/50, *v*/*v*) and pure IPA. The reconstituted standard mixtures were then analyzed together with the original standard mixtures as controls. Evaporation recovery was calculated by dividing the peak area of each analyte in the reconstituted samples by that in the untreated control.

#### 3.7.2. Limits of Detection and Quantification, Linearity, Ion Ratios and Carryover Rate

LODs and LLOQs were established according to the EURACHEM guide [50]. As such, calibration levels of 0, 0.0001, 0.0003, 0.001, 0.003, 0.01, 0.03, 0.1, 0.3, 1, 3, 10, 25 and 50 mg/L were prepared for all 3 sets of standards in neat solvents. The concentration of the lowest calibration standard showing a signal-to-noise ratio of at least 3 was set as the LOD, while a signal-to-noise ratio of at least 10 was required for the LLOQ. Due to unavoidable carryover at very low concentrations, several compounds showed peaks that did not increase in height at the next higher concentration level. In those cases, the LOD could not be determined, and the LLOQ was set to the lowest level at which the peak areas significantly increased and the signal-to-noise ratio was at least 10. The ULOQ was determined by visual inspection of the calibration curve. An r^2^ value greater than 0.995 was mandatory to determine linearity. Additionally, the determined ULOQ value needed to be within 95–105% of the actual concentration. LODs, LLOQs and ULOQs are provided in Supplementary Table S1. Ion ratios were obtained by averaging the peak area ratios of the qualifier and quantifier transition at three representative calibration levels within the linear range.

Analyte carryover was determined by injecting a pure solvent blank after each 1 mg/L of standard. For compounds with LLOQs higher than 1 mg/L, the peak area of the highest validation level was compared with that of the blank measured directly afterward. The carryover percentage was calculated as indicated in Equation (1):

Carryover [%] = Area (blank)/Area (standard) × 100(1)

#### 3.7.3. Determination of Apparent Recoveries and Repeatabilities in the Matrix

Apparent recoveries were evaluated using the standard addition method. As such, native concentrations of metabolites in pooled QC plasma were determined before spiking. Concentrations corresponding to 0.5, 1, 1.5, 2, 2.5 and 3 times the predetermined analyte concentration were spiked into the QC plasma. For metabolites < LOD in the QC sample, a concentration corresponding to 12.5 times the LLOQ (in pure solvents) was designated as the lowest spiking level of plasma. Additionally, spiking levels of 25, 37.5, 50, 62.5 and 75× LLOQ were prepared. The standard addition protocol consisted of drying down mixed standard solutions under a gentle stream of nitrogen, reconstituting the residue into 200 µL of plasma and 800 µL of cold IPA (−20 °C), vigorous vortexing, transfer to Eppendorf reaction tubes and centrifugation at 14,350× *g* at 4 °C for 10 min. One aliquot of the supernatants was further diluted 1:20 (*v*:*v*). For comparison, equal volumes of spiking solutions were processed in the same manner, except that 1 mL of IPA/water (80/20, *v*/*v*) was used for reconstitution of analytes.

Spiking was conducted in triplicate for each level. Two dilutions of plasma (1:5, 1:100, *v*:*v*) were prepared, and samples were measured by HILIC-MS/MS and RP-MS/MS as described above. For metabolites showing good linearity (r^2^ > 0.995), average R_As_ were calculated as the ratios of the slopes of the regression lines from the spiked samples and the standards in neat solvents. For analytes showing quadratic responses, R_As_ were calculated as indicated in Equation (2). The metabolite concentrations in the spiked samples (c_sp_) were calculated based on quadratic calibration curves established with pure solvent standards. Afterward, the concentration determined in the non-spiked sample (c_NS_) was subtracted, and the resulting difference was divided by the spiking concentration of the analyte (c_A_) at the respective level. The mean R_A_ was calculated by averaging the R_As_ obtained from six levels prepared and measured in triplicates (*n* = 18).

R_A_ [%] = (c_sp_ − c_NS_)/c_A_ × 100 (2)

To determine the repeatability, R_As_ were calculated at each spiking level using Equation (2). The repeatability was represented by the RSDs of the recoveries determined at six spiking levels in triplicate (*n* = 18).

### 3.8. Applicability to Plasma Samples from Different Animal Species

In order to test the applicability of the method, human, rat, bovine and chicken plasma samples were worked up as described above and analyzed. To minimize instrument bias, samples were measured in duplicate in a randomized sequence. The same two dilutions (1:5 and 1:100 in total) were measured to obtain the metabolite concentrations in animal samples. Recovery correction was applied only when the R_A_ values obtained from porcine plasma were below 70% or higher than 130%. In this case, measured analyte concentrations were corrected by the R_As_ according to Equation (3).

c _corrected_ = c _calculated from calibration curve_ × 100/R_A_ [%] (3)

To assess the method trueness, the metabolite concentrations in human plasma NIST SRM 1950 were compared to the certified values.

## 4. Conclusions

In this paper, we present the first fully validated, large-scale, targeted metabolomics method for the accurate and reliable quantification of porcine plasma metabolites. Two complementary LC techniques, namely RP and HILIC, were coupled to tandem mass spectrometry to cover 235 porcine plasma metabolites from 17 compound classes. Each analyte was determined using two SRM transitions to guarantee accurate identification. Fast polarity switching and sSRM were utilized to accelerate the methods and increase the throughput capacity, with a total analysis time of 39.5 min per sample. Sample preparation was simplified as a “dilute and shoot” approach, avoiding the derivatization of polar compound classes. The fully validated methods demonstrated minimal carryover, excellent linearity and repeatability and high accuracy. The inclusion of recovery data ensures robust quantification by correcting matrix effects and potential analyte losses during sample preparation [41]. The applicability test provides valuable concentration references for the validation and quantification of metabolites in plasma from other animal species. Future studies should focus on validating these methods in plasma from other animal species and/or other biological matrices. In conclusion, this study presents two comprehensively validated LC-MS/MS methods for the accurate quantification of 235 porcine plasma metabolites, setting a new standard for large-scale targeted metabolomics in porcine plasma.

## Figures and Tables

**Table 1:** Metabolite concentrations in NIST reference plasma and method trueness (concentrations determined by HILIC-MS/MS and RP-MS/MS relative to the NIST reference values).

Compound Name	c in NIST Reference Plasma (µg/L)	Measured c in NIST Plasma (µg/L)	Trueness (%)
Methionine	3325	2146	65
Leucine	13,158	9038	69
Proline	20,298	15,859	78
Valine	21,318	17,053	80
Threonine	14,219	11,915	84
Alanine	26,724	22,449	84
Serine	10,067	9218	92
Isoleucine	7273	6823	94
Tyrosine	10,373	9770	94
Phenylalanine	8364	8287	99
Lysine	20,400	23,637	116
Arginine	14,168	23,863	168
Creatinine	6789	7672	113
Urea	234,500	224,888	96
Cholesterol	1,514,000	645,655	43
Cholecalciferol	25		
Calciferol	1.0		

## Data Availability

Data are contained within the article and Supplementary Materials. Selected MS instrument files are available at https://doi.org/10.5281/zenodo.14652873, accessed on 15 January 2025. Additional inquiries can be forwarded to the corresponding author.

## Supplementary Materials

The following supporting information can be downloaded at: https://www.mdpi.com/article/10.3390/molecules30030706/s1, Table S1: Sensitivity, linearity and carryover of metabolites; Table S2: Apparent recoveries and repeatability. Compounds with R_As_ calculated based on quadratic or single-level calibration curves are marked with an asterisk; Table S3: Metabolite concentrations in plasma from diverse species; Table S4: Optimized sSRM transitions along with retention times and ion ratios; Table S5: Library of metabolites containing CAS numbers, sum formulas, SMILES codes, exact masses, log *p* values and providers.
